# Vertical Umbilical Incision Achieves Better Cosmesis Than Periumbilical Incision in Neonates and Infants

**DOI:** 10.7759/cureus.36589

**Published:** 2023-03-23

**Authors:** Hirofumi Tomita, Naoki Shimojima, Akihiro Shimotakahara, Ikkei Tamada, Miki Ishikawa, Makoto Hashimoto, Ayano Tsukizaki, Kazuaki Miyaguni, Seiichi Hirobe

**Affiliations:** 1 Surgery, Tokyo Metropolitan Children’s Medical Center, Tokyo, JPN; 2 Plastic and Reconstructive Surgery, Tokyo Metropolitan Children’s Medical Center, Tokyo, JPN

**Keywords:** children, cosmetic outcome, umbilical approach, umbilical incision, minimally invasive surgery

## Abstract

Purpose: The transumbilical approach is widely used for minimally invasive surgery in children. We compared herein the postoperative cosmesis between two types of transumbilical approach: a vertical incision versus periumbilical incision.

Methods: Patients with a transumbilical laparotomy before age one year were prospectively enrolled between January 2018 and December 2020. A vertical incision or periumbilical incision was chosen at the surgeon’s discretion. After excluding patients receiving a relaparotomy via another site, a questionnaire about the appearance of the umbilicus was completed by the patients’ guardians at postoperative month 6 to assess satisfaction and determine the visual analog scale score. A photograph of the umbilicus was taken while the questionnaire was being administered for later assessment by surgeons blinded to the scar and umbilical shape.

Results: Forty patients were enrolled; 24 patients received a vertical incision while 16 received a periumbilical incision. The incision length was significantly shorter in the vertical incision group (median: 2.0; range: 1.5-3.0 cm vs. median: 2.75; range: 1.5-3.6 cm) (p = 0.001). The patients’ guardians reported significantly higher satisfaction (p = 0.002) and higher scores on the visual analog scale (p = 0.046) in the vertical incision group (n = 22) than in the periumbilical incision group (n = 15). The surgeons’ evaluation was associated with significantly more patients with a vertical incision than with a periumbilical incision achieving a cosmetically preferable outcome, including an invisible or fine scar and a normal umbilical shape.

Conclusion: A vertical umbilical incision can provide better postoperative cosmesis than a periumbilical incision.

## Introduction

The transumbilical approach in laparotomy was first described in 1986. While it was originally used in pyloromyotomies in children with hypertrophic pyloric stenosis [1], it is now widely applied to children with various diseases, having come to represent a new paradigm for minimally invasive surgery [2]. The versatility of this technique lies in the fact that the umbilicus has a natural skin crease towards the center of the abdomen which hides the surgical scar, resulting in favorable postoperative cosmesis [3]. Although postoperative cosmesis is one of the main reasons for choosing the transumbilical approach in laparotomy, postoperative cosmetic outcomes have not been discussed in detail. We herein compared two types of transumbilical approach, one employing a vertical incision and the other employing a periumbilical incision, focusing on the cosmetic outcomes.

## Materials and methods

Patients and ethical consideration

Patients who underwent a transumbilical laparotomy before age one year were prospectively enrolled in the current observational study between January 2018 and December 2020. The type of umbilical incision (vertical or periumbilical) was chosen by the individual surgeon. One of the inclusion criteria was a transumbilical operation under direct vision; therefore, patients receiving pure laparoscopic surgery were excluded. The present study was approved by the ethics committee of Tokyo Metropolitan Children’s Medical Center (approval number: H29b-126), and written informed consent was obtained from the patients’ guardians.

Surgical technique: vertical incision

**Figure 1:**
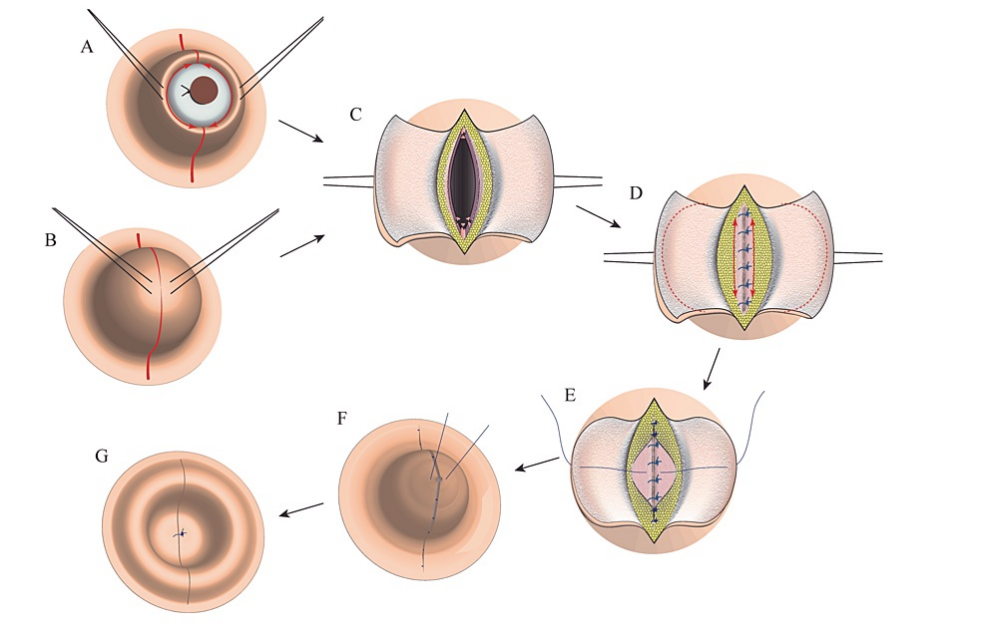
Transumbilical laparotomy via vertical incision followed by umbilicoplasty. Image credits: Hirofumi Tomita

Figure 1 shows the vertical umbilical incision followed by umbilicoplasty, a technique that we developed previously as an alternative to the conventional method. A vertical incision centering on the umbilicus was initially drafted at 1.5-3.0 cm in length, which usually exceeded the umbilical contour. Then, traction sutures were created bilaterally on the skin surrounding the umbilical cord remnant (Figure 1A) or at the bottom of the umbilicus if the patient had already achieved umbilical cord separation (Figure 1B). The skin was incised, and bilateral skin flaps were created by coring out the umbilical cord remnant. The abdominal cavity was explored via a vertical incision of the linea alba followed by resection of the umbilical cord remnant with ligation of the ductal remnants (Figure 1C). The subsequent abdominal procedure was determined according to the underlying disease; if necessary, the incision was elongated vertically. After completing the abdominal procedure, the fascia was closed longitudinally with 2-0 or 3-0 multifilament interrupted or continuous absorbable sutures (Figure 1D) followed by umbilicoplasty. To correct tugged skin on the sides of the umbilicus and form an oval umbilical fossa, the subcutaneous fat layers were detached from the fascia and sutured together on the cranial and caudal sides of the umbilicus; if needed, the skin flaps were trimmed to 1 cm in length (Figure 1 D-E). Subsequently, using an untied 5-0 absorbable multifilament suture, the top of the bilateral skin flaps was anchored to the fascia to form the umbilical bottom, and the flaps were longitudinally sutured together using dermal 5-0 absorbable multifilament sutures (Figure 1F). The skin flaps were everted and fixed to the fascia by tying the anchoring suture (Figure 1G). The umbilical bottom was compressed for two days using a cotton ball with ointment.

Surgical technique: periumbilical incision

Previous studies have described laparotomies via periumbilical incision [2-3]. According to the underlying disease, a semicircular, supra- or infra-umbilical incision was made along the umbilical contour. An omega-shaped extension of the incision was made if needed. The incision length was defined as the linear distance from one end of the incision to the other. The abdominal cavity was explored via a transverse incision of the linea alba and rectus abdominis. After completing the abdominal procedure, the muscle layer was closed transversely with 2-0 or 3-0 multifilament interrupted sutures. Finally, the skin was closed using subcutaneous and dermal 5-0 absorbable multifilament sutures.

Evaluation of cosmetic outcomes by the patients’ guardians

A questionnaire was completed at postoperative month 6 in the outpatient clinic by the patients’ guardians, who rated their satisfaction with the appearance of the umbilicus as “satisfied,” “uncertain,” or “dissatisfied.” Additionally, they rated the appearance of the umbilicus from 0 (the worst) to 100 (ideal) using the visual analog scale. In patients requiring a relaparotomy, those who underwent a relaparotomy via the same incision as in the first operation after elongation of the incision were evaluated for cosmetic outcomes while patients who underwent a relaparotomy via a different site were excluded.

Evaluation of cosmetic outcomes by surgeons

A photograph of the umbilicus was taken at postoperative month 6 while the questionnaire was being administered to the parents. The scar and umbilical shape in the photos were then reviewed and assessed by three, blinded surgeons who are also coauthors of the present report (IT, MI, and AT). The postoperative scar was assessed using a scale previously reported by Cozzi et al., in which “excellent” denotes a faint or non-evident scar, “good” denotes a fine and linear scar, “poor” denotes a visible, widened or atrophic scar, and “unacceptable” denotes an unsightly, visibly hypertrophic or hyperpigmented scar [4]. The umbilical shape was assessed in terms of whether the umbilical bottom was dented, flat or protruding; and whether the umbilical contour was regular or distorted. In case there was a disagreement among the assessments, the median value or the majority opinion was adopted.

Statistics

Categorical data were expressed as the number of the patients (%), and Chi square test or Fisher’s exact test was used for comparison. Continuous data were expressed as the median (range). Differences between the continuous data were analyzed using Welch’s t-test. p < 0.05 was considered to indicate statistical significance. All statistical analyses were performed using SPSS software, version 25.0 (IBM SPSS, Chicago, IL, USA).

## Results

Forty patients were enrolled, and a vertical incision was made in 24 patients while a periumbilical incision was made in 16. Of the patients with a periumbilical incision, three patients with Hirschsprung’s disease received an infraumbilical incision, and the remaining patients received a supraumbilical incision. Table 1 summarizes the patient characteristics. There was no significant difference between the vertical incision and periumbilical incision groups in terms of sex, age, body weight at surgery, disease, or preoperative umbilical status. All four patients presenting an umbilical hernia (three with Hirschsprung’s disease and one with intussusception) received a laparotomy via a vertical incision in the hernial orifice. Patients with hypertrophic pyloric stenosis were not included because pyloromyotomies for hypertrophic pyloric stenosis are performed purely laparoscopically at the study center. All the procedures were completed by pediatric surgeons alone (no plastic surgeons were involved). The procedures involving a vertical incision and those involving a periumbilical incision were performed by 10 pediatric surgeons with 1-12 years of experience and six pediatric surgeons with 1-10 years of experience, respectively.

**Table 1:** Patient characteristics.

	Vertical incision (n = 24)	Periumbilical incision (n = 16)	p value
Male sex	14 (58.3%)	11 (68.8%)	0.51
Age at operation (days)	7 (0–347)	4.5 (0–352)	0.72
Body weight at operation (kg)	3.2 (2.3–8.0)	3.3 (2.4–7.8)	0.55
Disease			0.39
Duodenal atresia/stenosis	3 (12.5%)	3 (18.8%)	
Intestinal atresia/stenosis	6 (25.0%)	2 (12.5%)	
Malrotation/intestinal volvulus	4 (16.7%)	7 (43.8%)	
Intestinal duplication	2 (8.3%)	-	
Intussusception	2 (8.3%)	1 (6.3%)	
Hirschsprung’s disease	5 (20.8%)	3 (18.8%)	
Ovarian cyst	2 (8.3%)	-	
Preoperative umbilical status			0.11
Umbilical cord stump	14 (58.3%)	7 (43.8%)	
Just after umbilical cord separation	1 (4.2%)	3 (18.8%)	
Normal appearance	5 (20.8%)	6 (37.5%)	
Umbilical hernia	4 (16.7%)	-	

Table 2 shows the operative findings and postoperative complications. The incision length was significantly shorter in the vertical incision group (p = 0.001). The vertical incision was made beyond the umbilical contour in a cranial and/or caudal direction in all 24 patients while an omega- shaped extension was made in 14 (87.5%) of 16 patients in the periumbilical incision group (p = 0.15). Laparoscopy use, operating time, and postoperative complications did not differ significantly between the groups. In the vertical incision group, four patients underwent a relaparotomy. Two of these patients had an intestinal volvulus, one had an intestinal obstruction, and one had anastomotic leakage. Two of the four patients in the vertical incision group underwent a relaparotomy with cranial elongation via the same incision as in the first operation. In the periumbilical incision group, two patients required a relaparotomy. Of these, one had a gastric perforation that had been overlooked and one had a biliary obstruction. The patient with the gastric perforation underwent a relaparotomy via the same incision as in the first laparotomy with transverse elongation.

**Table 2:** Operative findings and postoperative complications. *Intestinal volvulus (n=2), intestinal obstruction (n=1), anastomotic leakage (n=1); **Overlooked gastric perforation (n=1), biliary obstruction (n=1)

	Vertical incision (n = 24)	Periumbilical incision (n = 16)	p value
Incision length (cm)	2.0 (1.5–3.0)	2.75 (1.5–3.6)	0.001
Incision beyond the umbilical contours	24 (100%)	14 (87.5%)	0.15
Laparoscopy	8 (33.3%)	3 (18.8%)	0.47
Operating time (min)	147 (34–533)	135 (61–550)	0.85
Complications			
Wound infection	2 (8.3%)	1 (6.3%)	1.00
Incisional hernia	0 (0%)	2 (12.5%)	0.15
Granulation	1 (4.2%)	1 (6.3%)	1.00
Relaparotomy	4 (16.7%)*	2 (12.5%)**	1.00

**Figure 2:**
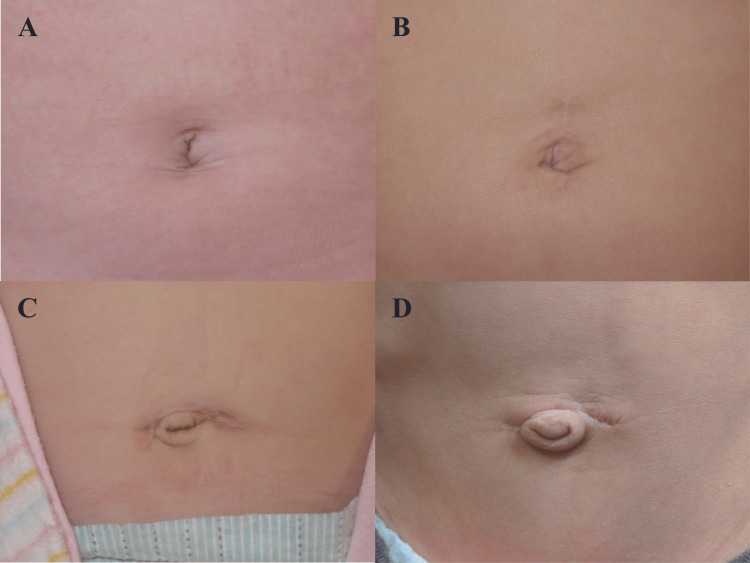
Postoperative photographs of the umbilical region at month 6 after transumbilical laparotomy. A: After vertical 2.0 cm incision for ovarian cyst at age 1 day; B: After vertical 2.5 cm incision for intussusception at age 9 months; C: After supraumbilical 2.0 cm incision for malrotation at age 9 days; D: After supraumbilical 3.0 cm incision for intussusception at age 11 months

Figure 2 shows the postoperative umbilical photographs. The vertical incision achieved significantly higher satisfaction (p = 0.002), with the results in all 21 (100%) patients evaluated as “satisfied” by the patients’ guardians. Meanwhile, the results in patients receiving a periumbilical incision were rated “satisfied” (n=9, 60.0%), “uncertain” (n=5, 33.3%) or “dissatisfied” (n=1, 6.7%). The visual analog scale for umbilical appearance was 88.5 (33-100) in patients receiving a vertical incision and 70 (9-100) in patients receiving a periumbilical incision, revealing a significant preference for the vertical incision (p = 0.046). The postoperative photograph was missing for one patient in the vertical incision group. The surgeons’ evaluation was associated with significantly more patients with a vertical incision than with a periumbilical incision achieving a cosmetically preferable outcome, including an invisible or fine scar and a normal umbilical shape. Tables 3-4 summarize the assessment results of the cosmetic outcomes. One patient who had undergone a laparotomy via a supraumbilical, 3.0 cm omega incision for intussusception at age 11 months received umbilicoplasty for a protruding umbilicus (Figure 2D) at postoperative month 17.

**Table 3:** Cosmetic outcomes evaluated using a questionnaire at postoperative month 6. *From 0 (the worst umbilicus) to 100 (ideal umbilicus)

	Vertical incision (n = 22)	Periumbilical incision (n = 15)	p value
Guardians satisfaction’			0.002
Satisfied	22 (100%)	9 (60.0%)	
Uncertain	-	5 (33.3%)	
Dissatisfied	-	1 (6.7%)	
Visual analog scale*	88.5 (33–100)	70 (9–100)	0.046

**Table 4:** Cosmetic outcomes evaluated by surgeons using a photograph at postoperative month 6.

	Vertical incision (n = 21)	Periumbilical incision (n = 15)	p value
Scar			<0.001
Excellent	13 (61.9%)	2 (13.3%)	
Good	8 (38.1%)	6 (40.0%)	
Poor	-	7 (46.7%)	
Unacceptable	-	-	
Umbilical shape			
Umbilical bottom			<0.001
Dented	16 (76.2%)	1 (6.7%)	
Flat	5 (23.8%)	11 (73.3%)	
Protruding	-	3 (20.0%)	
Umbilical contour			0.03
Regular	18 (85.7%)	7 (46.7%)	
Distorted	3 (14.3%)	8 (53.3%)	

## Discussion

Since Tan and Bianchi [1] first described transumbilical pyloromyotomy using a circum-supraumbilical incision, various modifications to the procedure have been suggested. Alberti et al. advocated making a semicircular incision in the right umbilical skin fold to overcome the difficulty of maneuvering a large pyloric mass through the incision during pyloromyotomy and reported a reduction in the risk of postoperative wound complications, such as infection and incisional hernia, which are sometimes associated with transumbilical surgery [5]. To improve postoperative cosmesis, Emil et al. performed pyloromyotomy via an infra-circumumbilical incision in a manner similar to that used in laparoscopic pyloromyotomy [6]. During surgery for miscellaneous abdominal pathologies requiring better abdominal access, Soutter et al. reported using a nearly circumferential (350°) incision [7] while Besson et al. and Lazar et al. proposed using a Y- or T-shaped skin incision with umbilicoplasty [8-9], and Ghaffarpour et al. described the so-called, U-u umbilicoplasty [10]. A periumbilical incision with an omega-shaped extension, as seen in the present study, has also been reported in previous studies [2-3, 11].

To date, only Cozzi et al. have scientifically assessed the cosmetic outcomes after a transumbilical laparotomy and reported that the parents of all 57 patients, who were interviewed by telephone, rated post-pyloromyotomy periumbilical cosmesis at five months to 15 years as excellent or good [4]. In addition, Cozzi et al. noted that 36 of 40 patients achieved excellent or good cosmetic outcomes whereas four had poor outcomes, and none of the patients had unacceptable outcomes in photographs assessed by five, blinded panel members [4]. In contrast to Cozzi et al.’s evaluation of the final appearance of the periumbilical scar, the present study assessed the postoperative appearance of the umbilicus using a questionnaire addressed to the patients’ guardians. Our results demonstrated that the vertical incision achieved significantly better cosmetic outcomes than the periumbilical incision. To the best of our knowledge, the present study is the first to compare cosmetic outcomes of different surgical approaches in transumbilical laparotomy for children. While in their evaluation of the surgical scars, Cozzi et al. reported satisfactory outcomes in 90% of the patients (36 of 40 patients had an outcome rated excellent or good), in the present study only 53.3% of the patients (8 of 15) in periumbilical incision group had a satisfactory outcome. The patients in the report by Cozzi et al. had hypertrophic pyloric stenosis, the most common indication for transumbilical laparotomy, which requires a relatively small incision. On the other hand, the diseases in our patients were varied, did not include pyloric stenosis, and requiring an omega-shaped elongation in most cases. The postoperative scar was unsightly, and the umbilical contour was distorted probably because the omega-shaped elongation is difficult to perform and reconstruct.

More recently, the intraumbilical incision, a circular incision made around the umbilical cord [12-13], transumbilical vertical incision [13-14], and umbilical Benz incision [15] have been developed. While a periumbilical incision leaves a scar around the umbilicus, an intraumbilical incision allows the surgeon to hide the scar in the folds in the umbilical bottom, resulting in less visible abdominal scars. In the present study, vertical scars exceeding the umbilical contour did not enlarge with the patient’s growth but instead showed a tendency to contract. Vertical incisions up to 2.0 cm in neonates were completely hidden by the umbilicus over time while the scar after an omega extension via periumbilical incision often became distorted and noticeable. In addition to minimal postoperative scarring, our vertical incision technique also has the benefits of allowing safe, direct access to the peritoneal cavity regardless of umbilical status. Especially in neonates, vertical incisions required a shorter incision length than periumbilical incisions because excision of the umbilical cord remnant provided a larger surgical view, one of the factors contributing to favorable cosmetic outcomes; subsequent umbilicoplasty was easy to perform; as a result, the operative times of the vertical incision and periumbilical incision groups were similar. Moreover, because our vertical incision technique tightly closed the umbilical ring (a natural defect in the fascia under the umbilicus originating from the umbilical cord), the risk of developing incisional hernia and a protruding umbilicus after transumbilical surgery was reduced, and the preexisting umbilical hernia was simultaneously able to be used for abdominal access before surgical closure. In contrast, because the periumbilical incision involved a transverse fascial incision near the umbilical ring, defects in the fascia were often worsened, and abdominal wall was weakened, resulting in a protruding umbilicus.

The chief limitation of the current study is the non-randomized study design; the procedures were chosen at the surgeons’ discretion, resulting in selection bias. Moreover, our patients were followed-up for only six months; therefore, further research, preferably a randomized control study, is needed to evaluate the long-term cosmesis.

## Conclusions

The present study demonstrated that a vertical umbilical incision combined with umbilicoplasty provided cosmetically better outcomes in neonates and young infants as evaluated by the patients' guardians than a periumbilical incision in a transumbilical laparotomy. The surgeons’ evaluation was associated with significantly more patients with a vertical incision than with a periumbilical incision achieving a cosmetically preferable outcome, including an invisible or fine scar and a normal umbilical shape. The advantages of the vertical umbilical incision over the periumbilical incision were the ability to hide the scar in the fold of the umbilical bottom, a larger surgical view, general applicability regardless of the umbilical status, such as umbilical hernia, and reduced risk of postoperative incisional hernia. Further research, preferably a randomized control study, is needed to evaluate the long-term cosmesis.
